# MMP-12在非小细胞肺癌中的研究进展

**DOI:** 10.3779/j.issn.1009-3419.2014.01.05

**Published:** 2014-01-20

**Authors:** 源 温, 莉 蔡

**Affiliations:** 150081 哈尔滨，哈尔滨医科大学附属肿瘤医院内科 Department of Medicine, Affiliated Tumor Hospital of Harbin Medical University, 150081 Harbin, China

**Keywords:** 肺肿瘤, 基质金属蛋白酶12, Lung neoplasms, Matrix metalloproteinases 12

## Abstract

肺癌是世界范围内最常见的恶性肿瘤之一，其中约80%为非小细胞肺癌，其发病机制至今尚未完全阐明。最近研究证实基质金属蛋白酶（matrix metalloproteinases, MMPs）的调节异常和过度表达与多种疾病相关，其中有研究显示基质金属蛋白酶家族中的基质金属蛋白酶12（MMP-12）参与了非小细胞肺癌的浸润和转移。在非小细胞肺癌组织中MMP-12的表达明显高于癌旁组织，且MMP-12对非小细胞肺癌患者的预后评估亦有重要意义。因此，本文将围绕MMP-12在非小细胞肺癌中的研究进行简要的总结。

## Abstract (en)

Lung cancer is one of the most common malignant tumors in the world wide. Non-small cell lung cancer (NSCLC) accounts for approximately 80% of lung cancers, its pathogenesis have not been elucidated. Recently, researches have shown that dysregulation and excessive activity of the matrix metalloproteinases (MMPs) has been associated with many pathologies. Some results suggest that the matrix metalloproteinases 12 (MMP-12) participates in the invasion and metastasis of NSCLC. The expression of MMP-12 in non-small cell lung cancer is significantly higher than in adjacent tissues. And studies have shown that the MMP-12 has important prognostic significance in NSCLC. Therefore this article will briefly summarize the research progresses of MMP-12 in NSCLC.

肺癌是世界范围内最常见的恶性肿瘤之一，居恶性肿瘤死亡的首位，5年生存率不到15%，其中约80%为非小细胞肺癌（non-small cell lung cancer, NSCLC）^[1-3]^。肺癌的发生是一多基因参与的病理过程，由于其侵袭的生物学性状和高度异质性，其发病机制至今尚未完全阐明。基质金属蛋白酶（matrix metalloproteinases, MMPs）是一个分泌家族，是高度保守的一组锌离子依赖性蛋白水解酶。MMPs在调节细胞生长、运动、组织形成和应对损伤方面起到关键作用，不仅能降解基质蛋白，还能限制特定蛋白的水解，包括生长因子、细胞因子、受体和粘附分子等^[4, 5]^。最近研究^[4, 6]^发现MMPs的调节异常和过度表达与多种疾病相关，如关节炎、动脉粥样硬化和肿瘤。MMP-12作为基质金属蛋白酶家族中的一员，有关它的研究越来越多，MMP-12可以水解弹性蛋白、Ⅳ型胶原蛋白、纤维连接蛋白、层粘蛋白及软骨胶硫酸盐等细胞外基质成分^[7, 8]^。并有一项研究^[9]^表明MMP-12可能参与一些恶性肿瘤的浸润、转移。

## 1 MMP-12的结构与功能

到目前为止，已经发现的MMP家族至少有24种^[10]^，MMP-12是MMP家族中的重要一员，是在吸烟者的肺泡巨噬细胞内发现的。在肺组织中，MMP-12主要在肺泡巨噬细胞表达，也可在人类支气管上皮细胞和气道平滑肌细胞表达^[11, 12]^。MMP-12由活化的炎症巨噬细胞分泌，最早是作为弹性水解金属蛋白酶被发现的。近几年来随着人们对MMP-12的基因和结构不断探索，发现它的过度表达与许多疾病有关，如肺气肿、骨关节炎、动脉粥样硬化、慢性阻塞性肺疾病（chronic obstructive pulmonary disease, COPD）^[13-16]^等。并且MMP-12还可以诱导骨髓细胞缺陷导致异常骨髓形成、免疫抑制和肺腺癌^[17]^。

MMPs是锌依赖性蛋白酶家族，可以降解细胞外基质和基底膜的所有成分。人类*MMP-12*基因定位于染色体11q22.3，cDNA全长1, 800 bp，包含1个由1, 410 bp碱基对组成的开放阅读框，*MMP-12*基因含有10个外显子和9个内含子。MMP-12的活化是以54KD的酶原形式分泌，经过一系列的水解过程后，产生几种有活性形式的酶。在体内MMP-12催化活性的一个主要表现是它可以激活其他的MMPs，如MMP-2和MMP-3，引起MMP-12水解的级联反应。

## 2 MMP-12表达的调节

MMP-12的调节主要包括转录水平的调节和抑制物的调节。

### 2.1 转录水平的调节

MMPs mRNA水平的上升或下降，可影响宿主细胞或肿瘤细胞合成和分泌酶原的数量。研究发现，白细胞介素-1β、肿瘤坏死因子-α、巨噬细胞集落刺激因子、血管内皮生长因子、血小板生长因子-BB都能明显提高巨噬细胞中MMP-12 mRNA的水平，而转化生长因子-β1可以抑制细胞因子介导的MMP-12 mRNA和蛋白表达以及MMP-12的活性，但细胞因子对MMP-12 mRNA的表达具有选择性。

### 2.2 抑制物的调节

MMP-12的抑制物主要包括金属蛋白酶组织抑制物（tissue inhibitor of metalloproteinase, TIMP）、巨球蛋白等。比较了解的TIMPs有4种：TIMP-1、TIMP-2、TIMP-3和TIMP4，其中TIMP1和TIMP2的研究较为深入。目前MMP抑制剂分为异羟肟酸盐类和非异羟肟酸盐类两大类。异羟肟酸盐类包括：巴马司他、马力马司他、普啉马司他和BAY12-9566。其中普啉马司他是一种可以特异性地抑制MMP-2和MMP-9的广谱MMPs抑制剂，而BAY12-9566是一种选择性的TIMPs，能够抑制MMP-2、MMP-9和MMP-3。非异羟肟酸盐类包括：新伐司他（Neovastat）和Tanomastat。新伐司他是一种从鲨鱼软骨中提取的天然MMPs抑制剂，可抑制MMP-2、MMP-9、MMP-12的活性，并能够拮抗血管内皮细胞生长因子受体（vascular endothelial growth factor receptor, VEGFR），是一种多功能抗肿瘤血管生成药。α2巨球蛋白对MMPs的抑制作用有广谱性，由于α2巨球蛋白是血浆中分子量最大的蛋白质，只有在炎症部位MMPs的抑制作用才能起到效果。尽管MMP抑制剂的临床研究由于其副作用、溶解度差和特异性低而受到阻碍^[18]^，但是最近一些动物实验研究^[19, 20]^表明，在慢性阻塞性肺疾病的肺组织内，MMP-12抑制剂能够抑制炎症改变和肺气肿。

## 3 MMP-12在NSCLC研究中的进展

MMP-12主要是由巨噬细胞、单核细胞等炎性细胞分泌的，并且在正常组织中仅有少量表达或不表达。近年来一些研究发现MMP-12能够在多种恶性肿瘤中呈现高表达，例如皮肤癌、胃癌、肺癌、肝癌等。但是目前对MMP-12具体的作用机制还不够明确，且MMP-12在不同恶性肿瘤中的表达意义也不尽相同。很多研究表明MMP-12能促进肿瘤的浸润、转移，如VAN Nguyen等^[21]^在直肠癌中发现MMP-12可能与疾病进展和癌细胞转移相关；Kerkeela^[9]^在皮肤基底细胞和鳞状细胞癌组织中发现MMP-12的mRNA表达水平明显比癌前病变高，且与肿瘤的分化和侵袭程度有关；在肝切除术后肝癌患者中的研究发现，MMP-12可能是总生存率和肿瘤复发一个可靠的预后标志物^[22]^。但也有一些研究认为MMP-12可以抑制肿瘤的形成，如Yang等^[23]^报道在结直肠癌肿瘤患者中MMP-12高表达预后好，并能降低肿瘤血管生成。

在NSCLC中，Hofmann等^[24, 25]^利用DNA微阵列技术筛选NSCLC的癌旁组织和癌组织差异表达基因，结果表明MMP-12的表达在癌组织中是癌旁组织的4.1倍，其基因分析显示MMP-12在正常组织中没有表达，免疫组化分析显示MMP-12仅在肿瘤细胞中表达，MMP-12高表达的NSCLC患者淋巴转移和原位复发的概率比MMP-12低表达者高。Kader等^[26]^通过微阵列分析显示MMP-12在肺鳞癌中出现过度表达。潘虹等发现MMP-12在肺鳞癌中的表达高于肺腺癌^[27]^，可能与肺鳞癌大多起源于段或段以上的气管或支气管有关，许多患者合并有慢性阻塞性肺疾病。肺组织中蛋白酶与抗蛋白酶系统失衡是肺癌产生的重要影响因素。吸烟可以引起肺部巨噬细胞中MMP-12的表达^[28]^。MMP-12在吸烟引起的慢性阻塞性肺疾病中具有关键作用，并且慢性阻塞性肺疾病与肺癌高度相关^[29]^。从临床研究看，MMP-12与NSCLC尤其是长期吸烟患者的早期癌症相关死亡有密切关系^[30, 31]^。Qu等^[31]^在人肺腺癌、肺鳞癌、肺气肿及正常肺组织中通过mRNA水平比较发现，MMP-12在肺腺癌、肺鳞癌及肺气肿中的表达水平比正常肺组织明显上调，还发现MMP-12在肺泡Ⅱ型细胞上皮细胞中高表达能直接引起肺部肿瘤。尽管MMP-12外显子多态性的功能尚不清楚，但是已经表明MMP-12预示着NSCLC患者的预后不好^[32]^。

## 4 结语

对于晚期的NSCLC患者，无论是放疗或综合治疗，虽然取得了一定的治疗效果，但就其疗效及生存率而言，不能令人满意。随着分子生物学技术的发展和完善，以及对肺癌发病机制等认识的日益深化，基因治疗作为一种高效性、特异性、靶向性强的治疗方法，越来越受到广大医生的重视，一系列的实验室结果和一部分临床实验显示肺癌的基因治疗有广阔的前景。目前关于MMPs的研究较多，尤其MMP-12参与肺癌演变过程备受关注，因此，MMP-12在NSCLC中的作用机制及其抑制剂的深入研究将是今后肺癌治疗的重要内容。
